# The Effects of COVID-19 Pandemic Policy on Social Needs Across the State of Kansas and Western Missouri: Paired Survey Response Testing

**DOI:** 10.2196/41369

**Published:** 2023-04-25

**Authors:** Dinesh Pal Mudaranthakam, Sam Pepper, Tanner Fortney, Alexander Alsup, Jennifer Woodward, Kevin Sykes, Elizabeth Calhoun

**Affiliations:** 1 Department of Biostatistics & Data Science University of Kansas Medical Center Kansas City, KS United States; 2 Department of Population Health University of Kansas Medical Center Kansas City, KS United States; 3 Department of Family Medicine and Community Health University of Kansas Medical Center Kansas City, KS United States; 4 Department of Otolaryngology-Head and Neck Surgery University of Kansas Medical Center Kansas City, KS United States

**Keywords:** social determinants of health, COVID-19, food assistance program, public health, quality of life, well-being, health disparity, health inequity, health policy, Kansas, social work, socioeconomic

## Abstract

**Background:**

Studying patients’ social needs is critical to the understanding of health conditions and disparities, and to inform strategies for improving health outcomes. Studies have shown that people of color, low-income families, and those with lower educational attainment experience greater hardships related to social needs. The COVID-19 pandemic represents an event that severely impacted people’s social needs. This pandemic was declared by the World Health Organization on March 11, 2020, and contributed to food and housing insecurity, while highlighting weaknesses in the health care system surrounding access to care. To combat these issues, legislators implemented unique policies and procedures to help alleviate worsening social needs throughout the pandemic, which had not previously been exerted to this degree. We believe that improvements related to COVID-19 legislature and policy have positively impacted people’s social needs in Kansas and Missouri, United States. In particular, Wyandotte County is of interest as it suffers greatly from issues related to social needs that many of these COVID-19–related policies aimed to improve.

**Objective:**

The research objective of this study was to evaluate the change in social needs before and after the COVID-19 pandemic declaration based on responses to a survey from The University of Kansas Health System (TUKHS). We further aimed to compare the social needs of respondents from Wyandotte County from those of respondents in other counties in the Kansas City metropolitan area.

**Methods:**

Social needs survey data from 2016 to 2022 were collected from a 12-question patient-administered survey distributed by TUKHS during a patient visit. This provided a longitudinal data set with 248,582 observations, which was narrowed down into a paired-response data set for 50,441 individuals who had provided at least one response before and after March 11, 2020. These data were then bucketed by county into Cass (Missouri), Clay (Missouri), Jackson (Missouri), Johnson (Kansas), Leavenworth (Kansas), Platte (Missouri), Wyandotte (Kansas), and Other counties, creating groupings with at least 1000 responses in each category. A pre-post composite score was calculated for each individual by adding their coded responses (yes=1, no=0) across the 12 questions. The Stuart-Maxwell marginal homogeneity test was used to compare the pre and post composite scores across all counties. Additionally, McNemar tests were performed to compare responses before and after March 11, 2020, for each of the 12 questions across all counties. Finally, McNemar tests were performed for questions 1, 7, 8, 9, and 10 for each of the bucketed counties. Significance was assessed at *P*<.05 for all tests.

**Results:**

The Stuart-Maxwell test for marginal homogeneity was significant (*P*<.001), indicating that respondents were overall less likely to identify an unmet social need after the COVID-19 pandemic. McNemar tests for individual questions indicated that after the COVID-19 pandemic, respondents across all counties were less likely to identify unmet social needs related to food availability (odds ratio [OR]=0.4073, *P*<.001), home utilities (OR=0.4538, *P*<.001), housing (OR=0.7143, *P*<.001), safety among cohabitants (OR=0.6148, *P*<.001), safety in their residential location (OR=0.6172, *P*<.001), child care (OR=0.7410, *P*<0.01), health care access (OR=0.3895, *P*<.001), medication adherence (OR=0.5449, *P*<.001), health care adherence (OR=0.6378, *P*<.001), and health care literacy (0.8729, *P*=.02), and were also less likely to request help with these unmet needs (OR=0.7368, *P*<.001) compared with prepandemic responses. Responses from individual counties were consistent with the overall results for the most part. Notably, no individual county demonstrated a significant reduction in social needs relating to a lack of companionship.

**Conclusions:**

Post-COVID-19 responses showed improvement across almost all social needs–related questions, indicating that the federal policy response possibly had a positive impact on social needs across the populations of Kansas and western Missouri. Some counties were impacted more than others and positive outcomes were not limited to urban counties. The availability of resources, safety net services, access to health care, and educational opportunities could play a role in this change. Future research should focus on improving survey response rates from rural counties to increase their sample size, and to evaluate other explanatory variables such as food pantry access, educational status, employment opportunities, and access to community resources. Government policies should be an area of focused research as they may affect the social needs and health of the individuals considered in this analysis.

## Introduction

When people think of health care, facilities such as hospitals, primary care clinics, and other direct health care services typically come to mind. The direct services provided by health care professionals at these institutions are generally considered to constitute the health care system in its entirety. However, this is only one piece of the health care system. An individual’s social needs outside of health care facilities also directly impact their health, and are important in understanding the causes of many health conditions and health disparities and in determining the actions society should take to improve overall health [1]. For example, obesity is influenced by the consumption of foods with poor nutritive value, a decrease in physical activity, and many other factors, which in turn lead to negative long-term health impacts [2,3].

One area of scientific research focuses on the neighborhood effect, which involves the interaction of social and environmental factors and how these interactions impact housing, crime, and violence [2]. The Robert Wood Johnson Foundation formed the Commission to Build a Healthier America, a nonpartisan group focused on improving public health outcomes for vulnerable populations, and released a report in 2014 comparing average life expectancy by county demonstrating wide variations in outcomes based on race/ethnicity, gender, income, and other factors [2,4]. A geospatial analysis showed that children with asthma residing in certain hotspot counties considered to be underserved have higher social vulnerabilities and are associated with longer hospital stay durations [5]. Another study found that social risk factors were associated with persistent functional disability caused by juvenile idiopathic arthritis [6].

Among the 105 counties in Kansas, United States, Wyandotte County is ranked 102nd according to county health rankings [7]. Wyandotte County residents are very diverse, with a distribution of 24.4% African American and 26.9% Latino [8]. Despite having one of the largest populations in the state (165,265 people), Wyandotte County is ranked among the lowest 25% of counties in Kansas regarding health outcomes and health factors [7]. Among adults, Wyandotte has a smoking rate of 20%, an obesity rate of 40%, a physical inactivity rate of 39%, and an uninsured rate of 17% compared to state averages of 17%, 36%, 26%, and 11%, respectively. Such vulnerable populations have been disproportionately impacted by the COVID-19 pandemic, creating the added burdens of stress and family challenges, both of which have impacted mental well-being [9].

The COVID-19 pandemic, declared by the World Health Organization on March 11, 2020, created challenges not only for the public health system but also for children and families suffering from housing and food insecurity [10]. COVID-19 had a different impact on each population group. For example, children experienced an increase in food insecurity due to the rising costs of food and reduced access to in-person school lunches [10]. In addition, people of color have experienced higher rates of COVID-19 infections, fatalities, and lower vaccination rates [11]. A lack of access to health care has been shown to cause food insecurity as health complications lead to or exacerbate the issue [12]. Lower-income households purchased shelf-stable processed foods and were more reliant on these types of foods than fresh food [13].

Income and education are other significant contributors to food insecurity, with lower-income families and those with lower educational attainment experiencing greater levels of insecurity [14]. Both income and education were also found to be major factors contributing to food security in Arkansas, which is one of the most food-insecure states in the United States [14]. Moreover, the COVID-19 pandemic exacerbated food insecurity for groups such as unemployed students [15,16].

During the first surge of the pandemic, an interview conducted with 20 nurses indicated that nearly every patient they were serving was a person of color, especially individuals of Hispanic ethnicity, who constituted a majority of the COVID-19 patients [17]. Many patients were suffering from untreated comorbidities, especially cases of diabetes and hypertension, that made patients more susceptible to the more serious outcomes associated with COVID-19 [17]. Some nurses also described cultural differences; for example, many Hispanic patients would not seek treatment when they began experiencing symptoms, and only sought medical attention when they became seriously ill at which time they were admitted directly to the intensive care unit (ICU) [17].

Differences in geographic location, income, education, and employment could explain a large amount of the disparity in vaccination coverage between Hispanic and white populations [18,19]. Models developed by Williams et al [20] indicated that health insurance in particular accounted for a 0.9 percentage-point disparity, and if the Hispanic population had a 13 percentage-point higher health insurance coverage to match that of white respondents, their predicted vaccination coverage would be an additional 1% higher. Their findings suggested that lack of insurance, unfamiliarity with the health care system, and not knowing that vaccines are provided free of charge may explain part of the vaccination coverage difference between these groups [20]. Compared with individuals identifying as white, minority groups such as Black, Asian, and Native American populations were more likely to have higher rates of ICU admissions, hospitalizations, and deaths when adjusted for age [21]. Research studies differ on the exact level of disparity; however, some minority groups experienced hospitalization rates that were nearly 9-times higher and infection rates that were 27-times higher than those of white individuals [21-23]. Based on data collected during the pandemic, life expectancy projections indicate that individuals of all races will experience a decreased life expectancy; however, Black and Latino individuals are expected to lose 2 and 3 years of life expectancy, respectively [24].

Social needs have a serious impact on a patient’s access to care and vulnerability to the COVID-19 virus; this causes disparities in health outcomes between counties within the United States. Access to healthy food, health care services, and willingness to accept COVID-19 vaccines play a significant role in population health outcomes. To help minimize the impact of COVID-19, the United States implemented several policies. Moratoriums on evictions were implemented nationwide [25,26], meal plans for children and vulnerable populations were expanded [27], and stimulus funds were made available to nearly all citizens. These policies may have prevented many families from suffering dire consequences associated with business closures and COVID-19–related medical expenses. These programs aimed to help alleviate the elevated social needs issues across the nation.

Despite the vast amount of research aimed at examining the effects of COVID-19 on human health in the United States, very little research has been conducted to examine the outcomes associated with funds provided to households to combat food insecurity, the moratorium implemented to prevent evictions, and other federal policies on the population. This is an important gap in the literature that should be addressed as socials impacts could play an important role in directing federal policy during the next pandemic.

With this in mind, the research question we set out to address is whether there is a significant difference in social needs before and after the COVID-19 pandemic declaration. Additionally, we wanted to explore how the social needs of individuals compare across counties. A notable county of interest is Wyandotte County, Kansas. Compared to other counties in the Kansas City metropolitan area, Wyandotte has a high poverty rate of 16.9%, a large minority population, and a high crime rate [28,29]. Therefore, we expected Wyandotte County to be impacted the most by COVID-19 and the resulting polices. If we can better understand how COVID-19 policies and programs have impacted individuals’ social needs, we will gain a better understanding of how to address social needs from both legislative and individual perspectives.

Our study thus aimed to illustrate the differences in social needs between populations living in the different counties throughout the state of Kansas and western Missouri. These counties represent a combination of rural and urban counties throughout the two states; however, our research focuses primarily on the regions of Johnson (Kansas), Jackson (Missouri), and Wyandotte (Kansas), since these jurisdictions are urban/suburban, are located near one another geographically, and are part of the same metropolitan area. The socioeconomic factors are also quite different among these jurisdictions, which may be a cause of disparities in patient responses and experiences.

## Methods

### Study Design

We used a survey developed at The University of Kansas Health System (TUKHS), which is a modified version of the validated social needs survey built from the Health Leads Toolkit [30]. The updates were specific to the separation of personal violence from community violence as those resources may differ [31]. The survey responses are used by the 12 social workers at TUKHS who aid patients who answer “yes” to any of the social needs questions. The data were retrieved from TUKHS informatics data warehouse HERON (aka. i2b2). The data set consists of patient demographic information along with the social needs responses. R studio with R version 4.1.2 was used to perform the descriptive and statistical analyses [32].

### Ethical Considerations

The study protocol was approved by the Institutional Review Board at the University of Kansas Medical Center (STUDY00148041).

### Data Sources

The primary data element utilized for this study was the patient-administered social needs survey, which is completed by patients during their primary care visit at TUKHS. The survey included 12 questions related to social needs, which are listed in Textbox 1.

**Items of the social needs survey.**

Question 1: In the last 12 months, did you ever eat less than you should because there wasn’t enough? (Food)

Question 2: In the last 12 months, has your utility company shut off your service for not paying your bill? (Utility)

Question 3: Are you worried that in the next 2 months, you may not have stable housing? (Housing)

Question 4: Are you afraid you might be hurt in your home by someone you know? (Safe home)

Question 5: Are you afraid you might be hurt in your apartment building or neighborhood? (Safe area)

Question 6: Do problems getting child care make it difficult for you to work or study? (Child care)

Question 7: In the last 12 months, have you needed to see a doctor, but could not because of cost? (Health care access)

Question 8: In the last 12 months, did you skip medications to save money? (Medication)

Question 9: In the last 12 months, have you ever had to go without health care? (Skip health care)

Question 10: Do you have problems understanding what is told to you about your medical conditions? (Health literacy)

Question 11: Do you often feel that you lack companionship? (Support)

Question 12: If you answered YES to any questions above, would you like to discuss help? (Need help in general)

Data (responses to the survey) were abstracted from the electronic medical record along with the zip code, city, and county of the patient on January 4, 2022. After deidentification of the patient information, data from patients not in Kansas or western Missouri were removed. This provided a longitudinal data set with 248,582 observations (820 from 2017, 47,877 from 2018, 58,376 from 2019, 74,618 from 2020, and 66,891 from 2021) representing the catchment area of TUKHS. In addition to the survey questions, 2020 county population estimates were obtained from the May 2021 Annual Resident Population Estimates of the US Census Bureau; population division area deprivation index by zip code was obtained from the 2019 release for both Kansas and Missouri, and 2013 Rural-Urban Continuum Code (RUCC) classifications were obtained from the update on December 10, 2020. RUCC codes 1, 2, and 3 were classified as urban, and codes 4, 5, 6, 7, 8, 9, and 10 were classified as rural [33,34].

### Data Analysis

For the pre-post COVID-19 analysis, the longitudinal data set was subset down to a paired-response data set for 50,441 individuals who had at least one response before March 11, 2020, and at least one response after. If an individual had multiple responses before the pandemic declaration, the response closest to March 11, 2020, was kept, and if an individual had multiple responses after, the most recent response was kept (ie, that closest to January 4, 2022). A contingency table and bar chart were constructed to explore the data responses. An overall pre-post composite score was calculated by adding the responses coded as yes=1 and no=0 across the 12 questions. The 3M Social Determinants of Health (SDoH) score in combination with individual metrics has been used to model health care utilization within a population; SDoH scores were highly impactful in these models when compared to the other variables [35]. Recent studies have proposed a similar approach, referring to these composite scores as polysocial risk scores or health indices [36,37]. A comprehensive risk score helps with a holistic approach to conduct statistical evaluation, addressing outliers or patients with enormous social needs. For example, patients with multiple social conditions would have a higher composite score compared with that of patients under relatively minimal social conditions such as a lack of child support. The composite score allows researchers to evaluate the impact of risk scores on patients’ health outcomes [37].

A Stuart-Maxwell marginal homogeneity test was run to compare the pre and post composite scores (relative to the pandemic announcement on March 11, 2020) across all counties. McNemar tests were then run to evaluate whether there is a statistically significant difference for each question when compared to paired pre-post responses across all counties. County-level McNemar tests were performed for questions 1, 7, 8, 9, and 10 for the county groupings of Cass (Missouri), Clay (Missouri), Jackson (Missouri), Johnson (Kansas), Leavenworth (Kansas), Platte (Missouri), Wyandotte (Kansas), and Other.

## Results

**Figure 1:**
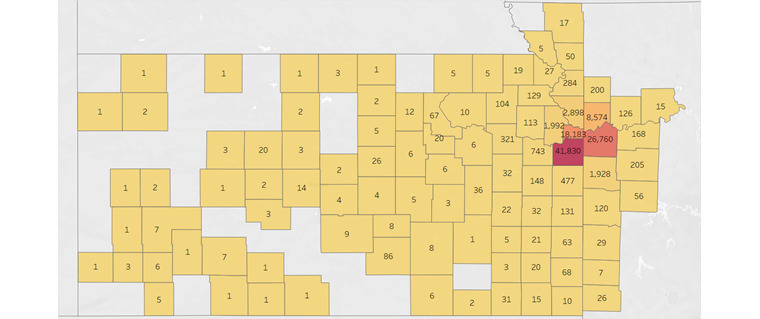
Heat map of the raw response count by counties under The University of Kansas Cancer Center catchment area; yellow indicates lower counts and red indicates higher counts. ©Mapbox ©OpenStreetMap.

**Figure 2:**
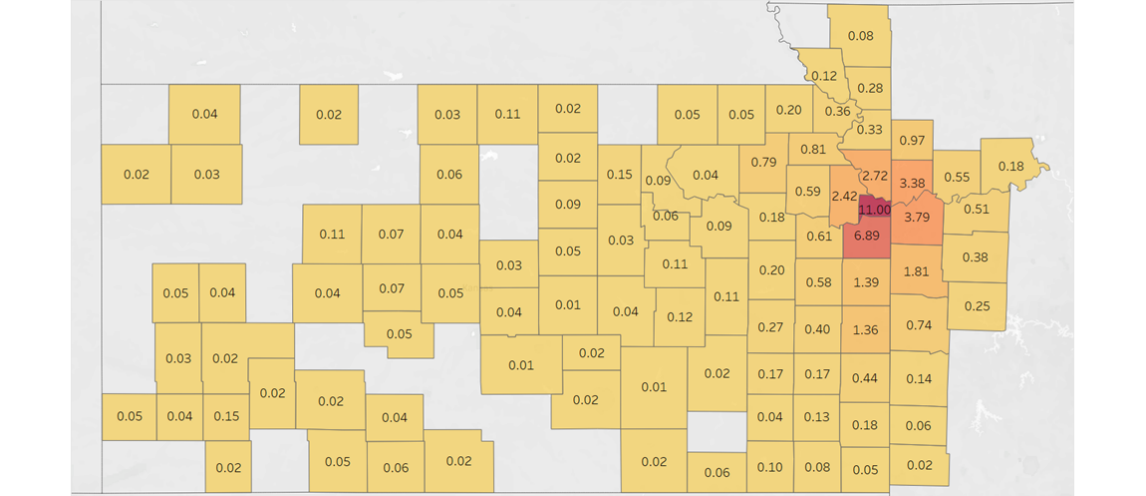
Heat map of the percentage of the total county population to responses by county in The University of Kansas Cancer Center catchment area; yellow indicates lower counts and red indicates higher counts. ©Mapbox ©OpenStreetMap.

The most recent responses were divided into a subset and the data set was visualized to determine the number of respondents per county. Figure 1 shows that the majority of patients in the data set reside in the immediate surrounding counties of TUKHS. The number of patients by county was then divided by their corresponding 2020 census county population estimate to determine the percentage of the county captured. Figure 2 shows that when adjusting for county populations, most of the respondents in the data set were still based in the immediate surrounding counties of TUKHS. Because of the low percentage of counties not immediately near TUKHS, we decided to classify the respondents into the counties of Cass, Clay, Jackson, Johnson, Leavenworth, Platte, Wyandotte, and Other, which created groupings with at least 1000 responses in each.

The yes/no counts by group for the most recent responses are provided in Table 1, showing that Wyandotte County consistently had a higher proportion of “yes” responses when compared to the other counties. Table 1 also shows that the proportion of “yes” response was below 6% for the most recent responses, demonstrating the rarity of this response. The county buckets of Cass, Clay, Jackson, Johnson, Leavenworth, and Platte all had a “yes” response rate below 4%.

The Stuart-Maxwell test showed a significant (*P*<.001) difference in the composite scores between the pre and post responses. Unadjusted *P* values of the McNemar test for each of the questions across all counties showed significance at an α level of .05 for all the questions except question 11 (Support). However, it should be noted that statistical significance does not necessarily mean that a meaningful difference exists given the rarity in the event of a respondent answering “yes.” This scenario is particularly apparent for questions 2 (Utility), 3 (Housing), 4 (Safe home), 5 (Safe area), and 6 (Child care). Therefore, questions 1 (Food), 7 (Health care access), 8 (Medication), 9 (Skip health care), and 12 (Need help in general) are the notable questions to consider further (Table 2).

Notable results from the county-level McNemar tests were related to questions 1, 7, 8, and 9 from Wyandotte County, which had a relatively high percentage of respondents who answered “yes” in the pre-COVID-19 category and subsequently answered “no” in the post-COVID-19 category. The unadjusted *P* values from the McNemar test were significant for these questions. When looking at the McNemar test results by county, significant reductions in respondents answering “yes” for question 1 were found in Clay, Jackson, Johnson, Wyandotte, and Other counties. Significant reductions in respondents answering “yes” for question 7 were found in Cass, Clay, Jackson, Johnson, Platte, Wyandotte, and Other counties. Significant reductions in respondents answering “yes” for question 8 were found in Cass, Clay, Jackson, Johnson, Platte, Wyandotte, and Other counties. Significant reductions in respondents answering “yes” for question 9 were found in Jackson, Johnson, and Wyandotte. Finally, no county buckets demonstrated a significant change in the response to question 10 (Table 2).

**Table 1:** Response distribution by counties with a minimum of 1000 patient responses.

Question^a^		Cass, MO^b^	Clay, MO	Jackson, MO	Johnson, KS^c^	Leavenworth, KS	Platte, MO	Wyandotte, KS	Other
**1: Food, n (%)**									
	No	1837 (98.29)	8235 (98.32)	25,655 (98.01)	36,331 (98.74)	1717 (97.95)	27,535 (98.78)	15,088 (95.63)	3897 (96.82)
	Yes	32 (1.71)	<25 (1.68)	520 (1.99)	463 (1.26)	36 (2.05)	34 (1.22)	689 (4.37)	128 (3.18)
**2: Utility, n (%)**									
	No	1853 (99.09)	8322 (99.31)	25,905 (98.95)	36,621 (99.42)	1741 (99.20)	2773 (99.36)	15,324 (97.01)	3961 (98.48)
	Yes	<25 (0.91)	58 (0.69)	275 (1.05)	215 (0.58)	<25 (0.80)	<25 (0.64)	473 (2.99)	61 (1.52)
**3: Housing, n (%)**									
	No	1859 (99.41)	8270 (98.62)	25,820 (98.62)	36,523 (99.15)	1723 (98.23)	2763 (99.03)	15,363 (97.26)	3945 (98.09)
	Yes	<25 (0.59)	110 (1.31)	360 (1.38)	313 (0.85)	31 (1.77)	27 (0.97)	432 (2.74)	77 (1.91)
**4: Safe home, n (%)**									
	No	1863 (99.68)	8367 (99.86)	26,115 (99.76)	36,755 (99.81)	1751 (99.77)	2784 (99.78)	15,739 (99.65)	4003 (99.53)
	Yes	<25 (0.32)	<25 (0.14)	62 (0.24)	70 (0.19)	<25 (0.23)	<25 (0.22)	55 (0.35)	<25 (0.47)
**5: Safe area, n (%)**									
	No	1867 (99.84)	8347 (99.59)	25,963 (99.20)	36,720 (99.72)	1747 (99.54)	2780 (99.64)	15,593 (98.73)	3988 (99.15)
	Yes	<25 (0.16)	34 (0.41)	210 (0.80)	102 (0.28)	<25 (0.46)	<25 (0.36)	201 (1.27)	34 (0.85)
**6: Child care, n (%)**									
	No	1857 (99.46)	8331 (99.49)	26,044 (99.54)	36,649 (99.57)	1740 (99.20)	2772 (99.50)	15,603 (98.93)	3989 (99.35)
	Yes	<25 (0.54)	43 (0.51)	120 (0.46)	160 (0.43)	<25 (0.80)	<25 (0.50)	168 (1.07)	26 (0.65)
**7: Health care access, n (%)**									
	No	1819	8069	25,160	36,045	1703	2722	14,945	3865
	Yes	49 (2.62)	306 (3.65)	1016 (3.88)	792 (2.15)	51 (2.91)	69 (2.47)	842 (5.33)	153 (3.81)
**8: Medication, n (%)**									
	No	1830 (97.38)	8134 (97.08)	25,410 (97.09)	36,179 (98.22)	1707 (97.21)	2733 (97.92)	15,102 (95.64)	3868 (96.17)
	Yes	40 (2.14)	245 (2.92)	761 (2.91)	655 (1.78)	49 (2.79)	58 (2.08)	689 (4.36)	154 (3.83)
**9: Skip health care, n (%)**									
	No	1849 (98.93)	8275 (98.76)	25,750 (98.39)	36,466 (99.01)	1726 (98.35)	2765 (99.10)	15,173 (96.10)	3899 (97.01)
	Yes	<25 (1.07)	104 (1.24)	421 (1.61)	364 (0.99)	29 (1.65)	25 (0.90)	616 (3.90)	120 (2.99)
**10: Health literacy, n (%)**									
	No	1837 (98.24)	8227 (98.19)	25,692 (98.17)	36,198 (98.30)	1698 (96.75)	2752 (98.71)	15,063 (95.44)	3876 (96.39)
	Yes	33 (1.76)	152 (1.81)	479 (1.83)	627 (1.70)	57 (3.25)	36 (1.29)	719 (4.56)	145 (3.61)
**11: Support, n (%)**									
	No	1805 (98.31)	7992 (98.19)	24,685 (96.19)	35,376 (97.00)	1674 (96.60)	2676 (97.24)	14,631 (94.66)	3718 (94.44)
	Yes	31 (1.69)	291 (3.51)	979 (3.81)	1094 (3.00)	59 (3.40)	76 (2.76)	826 (5.34)	219 (5.56)
**12: Need help in general, n (%)**									
	No	1807 (98.74)	8155 (98.19)	25,068 (97.64)	35,538 (98.52)	1690 (97.52)	2714 (98.44)	14,791 (95.98)	3790 (96.91)
	Yes	23 (1.26)	150 (1.81)	607 (2.36)	535 (1.48)	43 (2.48)	43 (1.56)	620 (4.02)	121 (3.09)

^a^For detailed questions, see Textbox 1.

^b^MO: Missouri.

^c^KS: Kansas.

**Table 2:** McNemar test results for responses before and after the COVID-19 pandemic declaration overall and by county.

Question^a^		OR^b^ (95% CI)	*P* value
**Q1: Food**			
	Overall	0.4073 (0.3577-0.4629)	<.001
	Cass, MO^c^	0.3636 (0.0844-1.2272)	.12
	Clay, MO	0.2381 (0.1259-0.4230)	<.001
	Jackson, MO	0.4038 (0.3106-0.5210)	<.001
	Johnson, KS^d^	0.4365 (0.3348-0.5652)	<.001
	Leavenworth, KS	1.6000 (0.4615-6.2161)	.58
	Other	0.4375 (0.2157-0.8435)	.02
	Platte, MO	0.4500 (0.1804-1.0339)	.06
	Wyandotte, KS	0.4014 (0.3201-0.5006)	<.001
Q2: Utility (Overall)		0.4538 (0.3853-0.5331)	<.001
Q3: Housing (Overall)		0.7143 (0.6183-0.8245)	<.001
Q4: Safe home (Overall)		0.6148 (0.4548-0.8263)	<.001
Q5: Safe area (Overall)		0.6172 (0.5029-0.7555)	<.001
Q6: Child care (Overall)		0.7410 (0.5820-0.9412)	.01
**Q7: Health care access**			
	Overall	0.3895 (0.3498-0.4331)	<.001
	Cass, MO	0.1667 (0.0420-0.4851)	<.001
	Clay, MO	0.4359 (0.3074-0.6106)	<.001
	Jackson, MO	0.4091 (0.3346-0.4980)	<.001
	Johnson, KS	0.4441 (0.3632-0.5409)	<.001
	Leavenworth, KS	0.5333 (0.1958-1.3400)	.21
	Other	0.3148 (0.1711-0.5515)	<.001
	Platte, MO	0.3684 (0.1844-0.6956)	.001
	Wyandotte, KS	0.3158 (0.2484-0.3985)	<.001
**Q8: Medication**			
	Overall	0.5449 (0.4928-0.6021)	<.001
	Cass, MO	0.2857 (0.1125-0.6434)	.001
	Clay, MO	0.6735 (0.4853-0.9295)	.02
	Jackson, MO	0.5464 (0.4492-0.6627)	<.001
	Johnson, KS	0.5076 (0.4186-0.6138)	<.001
	Leavenworth, KS	1.3000 (0.5266-3.3119)	.68
	Other	0.5556 (0.3265-0.9255)	.02
	Platte, MO	0.4815 (0.2281-0.9658)	.04
	Wyandotte, KS	0.5451 (0.4441-0.6671)	<.001
**Q9: Skip health care**			
	Overall	0.6378 (0.5574-0.7291)	<.001
	Cass, MO	0.2857 (0.0290-1.5006)	.18
	Clay, MO	1.0769 (0.4929-1.9118)	.89
	Jackson, MO	0.5436 (0.4095-0.7173)	<.001
	Johnson, KS	0.6825 (0.5127-0.9050)	.01
	Leavenworth, KS	0.7778 (0.2462-2.3470)	.80
	Other	0.7419 (0.4131-1.3144)	.34
	Platte, MO	0.7500 (0.2791-1.9394)	.66
	Wyandotte, KS	0.6068 (0.4819-0.7615)	<.001
**Q10: Health literacy**			
	Overall	0.8729 (0.7823-0.9737)	.02
	Cass, MO	0.6667 (0.2678-1.5863)	.42
	Clay, MO	0.7458 (0.4929-1.1208)	.17
	Jackson, MO	0.9125 (0.7241-1.1492)	.46
	Johnson, KS	0.9510 (0.7772-1.1633)	.65
	Leavenworth, KS	1.1000 (0.4241-2.8891)	>.99
	Other	0.7879 (0.4525-1.3583)	.44
	Platte, MO	0.7857 (0.3228-1.8630)	.69
	Wyandotte, KS	0.8263 (0.6729-1.0137)	.07
Q11: Support (Overall)		0.9337 (0.8583-1.0157)	.11
Q12: Need help in general (Overall)		0.7368 (0.6619-0.8198)	<.001

^a^For detailed questions, see Textbox 1.

^b^OR: odds ratio.

^c^MO: Missouri.

^d^KS: Kansas.

## Discussion

### Principal Findings

An interesting takeaway from the pre-post COVID-19 comparison is that the post-COVID-19 responses across the questions showed an improvement in social needs. Less respondents answered “yes” to the social needs questions in the post-COVID-19 survey period. At this point, we can only speculate on the reasons for this improvement. One possibility is that this was due to the government policies issued during the pandemic [38]. Across society, changes were made regarding economic relief, food availability, and the ability to evict tenants; thus, it is possible that these changes are directly impacting individuals’ social needs [39,40]. This trend was maintained when breaking down the analysis to the county level, notably in Wyandotte County. Based on the results, it is possible that Wyandotte County was affected by COVID-19 differently than Johnson County or Jackson County. It certainly appears that some of the responses to the social needs survey improved in the post-COVID-19 period to the greatest extent in Wyandotte County. Further investigation should be performed with respect to Wyandotte County to try and explain this observation. When looking at the most recent responses, Wyandotte County ranked higher in the proportion of “yes” responses to all questions except questions 4 and 11, and differences in outcomes were also significant when comparing all counties in the analysis.

Among the counties examined in this analysis, Johnson County and Jackson County have the two largest populations; however, Johnson County had the lowest affirmative response rate for nearly every question, while Jackson County had an affirmative response rate second only to that of Wyandotte County. This suggests that a county’s population is not proportional to the resources available for its residents to weather a pandemic-related storm and that large communities may have worse social needs outcomes than smaller communities. Similarly, both Johnson County and Jackson County are urban communities and yet their outcomes are strikingly different, suggesting that the urban/suburban population density may not be a factor in the social needs for a given population. The primary difference may lie in the socioeconomic factors associated with the various counties. Johnson County is a more socially affluent community with respect to nearly all metrics, with low unemployment rates, a strong social safety net, and good access to health care and educational opportunities. These factors should be considered in future research.

As observed in this study, the usage and expansion of assistance programs may lead to a reduction in social needs issues. Across the state of Kansas and in western Missouri, we found improvements in TUKHS survey responses after COVID-19 policies had been implemented. When broken down by county, it became clear that these programs impacted each county differently. As a society, we must better utilize assistance programs and policies that are in place and understand the different and unique needs of our diverse populations. Health and social workers should be well versed and keep their patients informed on programs that they are eligible for. These programs are not useful without public knowledge of their existence.

### Limitations

This study has several important limitations that should be considered when implementing policy recommendations and determining future research. First, the data are limited only to patients of TUKHS, located in the Kansas City Metropolitan Statistical Area. Over 98% of the unique responses were from individuals residing in counties that are part of the metropolitan area and this population may not be representative of other counties within the states of Kansas or Missouri. Due to socioeconomic conditions prevalent in rural areas, such as high rates of poverty, unemployment, and lack of social services, patients located in these counties may not see similar rates of improvements in social needs following the COVID-19 pandemic declaration.

Second, due to the large sample size and unadjusted *P* values, even small differences in outcomes will be statistically significant, but these results are not necessarily practically significant. A determination should be made as to what level of improvement is considered *practically* significant before policy recommendations can be made using this analysis. In addition, the sample size is much smaller for rural counties (<2% of responses), which could skew the results, and larger sampling would potentially correct for the rural-urban disparity.

Third, the data set does not include uninsured patients. Although we do not have data on household income for the individuals that responded to this survey, the presence of insurance could be used as a proxy variable, whereas those with insurance have higher incomes than those who do not. Because this study only considered those with insurance, we can presume that these individuals are less likely to answer “yes” to the questions presented in the survey, which could underrepresent the baseline scores as well as the level of improvement.

Moreover, the economic and societal hardships brought about by COVID-19 could have changed public perceptions of social needs. What was once considered an issue before the pandemic may not have been considered an issue after. Perhaps a societal shift in the perception or willingness to share social needs accounts for our findings. Patients may be less likely to disclose their social needs to health care professionals now that they have seen the government willingly address them. Perhaps the social needs survey was distributed with less importance during the pandemic as COVID-19 screening surveys took priority during patient visits. Survey responses are a subjective measurement that can be impacted by self-reported biases, as each respondent has different thoughts, behaviors, and feelings in the context of social needs. Some of their social needs could be relative to their perception based on past encounters.

Due to the limitations of the data and disparities in the patient population, the generalizability of this study may be limited to populations in urban and suburban areas. However, despite the small sample size, significant differences were found when aggregate patient responses from rural counties were compared to the urban counties, and previous research on rural poverty and lack of health care in these areas suggest a larger sample will not likely change the results [41]. By contrast, additional data from rural patients may increase the disparity in these populations and confirm the differences we identified.

### Policy Implications and Future Research

Further research should focus on certain counties, particularly those in rural areas to increase the number of patients from these areas. Since education has been identified as an important indicator of poverty and income, exploring educational programs in poor-performing counties could improve outcomes. Social workers and navigators play an important role in helping individuals access food pantries, educational opportunities, employment, and other resources that influence these outcomes; thus, further study should be directed toward outcomes associated with interactions that reduce barriers to care, and unit-level acuity metrics should be evaluated and reviewed frequently to understand the correlation between health outcomes and patient success rates in accessing these resources [42].

Finally, unlike the responses to many viruses that have spread throughout the world in recent decades, the United States took unprecedented steps to not only curtail the spread of COVID-19 but also to minimize the economic impact on the country and ensure the financial well-being of its people. Although it is unclear whether the country would implement similar policies in the event of a future pandemic, the impact of these policies should be thoroughly analyzed in the context of social needs outcomes, as they could be a significant reason why many individuals are not experiencing severe negative impacts.

## Abbreviations

ICU: intensive care unit
RUCC: rural-urban continuum code
SDoH: Social Determinants of Health
TUKHS: The University of Kansas Health System

## Data Availability

Detailed data cannot be shared publicly to protect the privacy of individual participants. Information to support the findings of these analyses can be made available by contacting the corresponding author, who, upon reasonable request and understanding of the intended use of the data, will provide the requested information in a manner that continues to protect individual patient information.
